# A novel benign and malignant classification model for lung nodules based on multi-scale interleaved fusion integrated network

**DOI:** 10.1038/s41598-024-79058-y

**Published:** 2024-11-11

**Authors:** Enhui Lv, Xingxing Kang, Pengbo Wen, Jiaqi Tian, Mengying Zhang

**Affiliations:** School of Medical Information & Engineering, Xuzhou Medical University, Xuzhou, Jiangsu China

**Keywords:** Lung nodule classification, Deep integration network, Lightweight network, Multi-scale learning, Biological techniques, Computational biology and bioinformatics

## Abstract

One of the precursors of lung cancer is the presence of lung nodules, and accurate identification of their benign or malignant nature is important for the long-term survival of patients. With the development of artificial intelligence, deep learning has become the main method for lung nodule classification. However, successful deep learning models usually require large number of parameters and carefully annotated data. In the field of medical images, the availability of such data is usually limited, which makes deep networks often perform poorly on new test data. In addition, the model based on the linear stacked single branch structure hinders the extraction of multi-scale features and reduces the classification performance. In this paper, to address this problem, we propose a lightweight interleaved fusion integration network with multi-scale feature learning modules, called MIFNet. The MIFNet consists of a series of MIF blocks that efficiently combine multiple convolutional layers containing 1 × 1 and 3 × 3 convolutional kernels with shortcut links to extract multiscale features at different levels and preserving them throughout the block. The model has only 0.7 M parameters and requires low computational cost and memory space compared to many ImageNet pretrained CNN architectures. The proposed MIFNet conducted exhaustive experiments on the reconstructed LUNA16 dataset, achieving impressive results with 94.82% accuracy, 97.34% F1 value, 96.74% precision, 97.10% sensitivity, and 84.75% specificity. The results show that our proposed deep integrated network achieves higher performance than pre-trained deep networks and state-of-the-art methods. This provides an objective and efficient auxiliary method for accurately classifying the type of lung nodule in medical images.

## Introduction

Lung nodules are potential precursors to lung cancer, the leading cause of death worldwide. Early detection, screening, and classification of these nodules are critical to reducing lung cancer mortality^1^. With the rapid development of detection technology, computed tomography (CT) technology has been widely used in the early clinical diagnosis of lung nodules^2^. Accurately assessing the characteristics of lung nodules from CT images remains a formidable challenge, given the complex nature of nodules and the inherent limitations of CT imaging technology. Therefore, it is highly desirable to investigate a reliable computer-aided diagnostic method for the accurate identification of benign and malignant nodules and early diagnosis of lung nodules.

Currently, deep convolutional networks have demonstrated remarkable proficiency in lung CT image recognition tasks. By automatically extracting high-level features from lung CT images, these networks enable highly accurate risk stratification predictions for lung nodules, thereby showcasing their distinct advantage in this field. For example, Biradar et al.^3^ proposed a 2D convolutional neural network to detect and classify lung nodules by extracting rich discriminative features from alternately stacked convolutional layers to capture the heterogeneity of lung nodules, achieving a classification accuracy of 88.76% on Kaggle CT scans. Dodia et al.^4^ proposed a novel deep learning architecture RFR V-Net for lung nodule classification. The method first uses receptive regularization in the convolutional layer of the V-Net network, and then uses a novel combination of SqueezeNet^5^ and ResNet^6^ for lung nodule classification. Lin et al.^7^ proposed a 3D VGG + ResCon network to mine the vertical information of lung cancer CT images, which accelerated the training efficiency of the model. In addition, after augmenting the dataset, the focal loss is replaced by the traditional cross-entropy loss to solve the problem of uneven distribution of positive and negative samples in medical data. The method achieved a classification accuracy of 93.62% on the LUNA16 dataset. Due to the small size of lung nodules and their rich 3D information, Yu et al.^8^ proposed a 3D Res U-Net for lung nodule classification. Yang et al.^9^ proposed a 3D multi-view convolutional neural network to capture spatial features and learn the spatial heterogeneity of lung nodules, achieving an accuracy of 96.04% on the LIDC dataset. However, the success of deep convolutional networks heavily relies on a large number of parameters and carefully annotated data. Typically, the data in the medical image domain is not as extensive as that in the natural image domain, with some publicly available medical datasets typically containing only a few thousand or even a few hundred medical images. Although the deep learning models described above achieve good network performance in lung nodule analysis and diagnosis, deep convolutional networks often show performance degradation when dealing with new data from the problem domain^10^.

Accordingly, many existing studies in medical image analysis have employed computer vision techniques to address performance degradation, such as deep network complexity reduction^11^, and data enhancement strategies^12^. Essentially, techniques aimed at reducing network complexity and enhancing data primarily concentrate on the designated task within a specific dataset. However, as of now, datasets containing pathologically confirmed lung nodules are significantly scarce and exhibit a high degree of imbalance in the distribution of benign and malignant cases. Consequently, there are limitations to enhancing model performance solely through refining the model or augmenting these imbalanced datasets^13^. To overcome this problem, a strategy known as transfer learning proposes that the features acquired for solving one task can also be beneficial for tackling tasks within other domains. For example, Da et al.^14^ investigated the performance of deep migration learning in lung nodule classification using a pre-trained model. When implementing the convolutional neural network model, the last layer is removed from the model and the output of the remaining model is adjusted to a one-dimensional vector. Therefore, the new model only performs feature extraction on the input data and no longer performs classification. Xie et al.^15^ proposed a knowledge-based multi-view collaborative deep model to learn a migratory ResNet-50 network to classify benign and malignant nodules using limited lung CT data and achieved 91.6% accuracy on LIDC. Shi et al.^16^ proposed a semi-supervised deep transfer learning (SDTL) framework for the diagnosis of benign and malignant lung nodules. The method first uses a pre-trained classification network to discriminate between lung nodules and nodular tissue, and then proposes a semi-supervised method based on iterative feature matching to utilize large available datasets without pathology results. In this study, Shi collected a total of 3,038 lung nodules with pathologically proven benign or malignant labels and 14,735 unlabelled nodules. Huang et al.^17^ proposed a self-supervised transfer learning (SSTL-DA) 3D CNN framework based on domain adaptation for benign and malignant lung nodule classification. This method first developed an adaptive slice selection to eliminate redundant noise in lung nodule samples; Then, a self-supervised learning network is constructed to learn robust image representations from CT images; Finally, a domain adaptation-based transfer learning method was designed to obtain discriminative features for classification, achieving an accuracy of 91.07% on the LIDC dataset. Shamrat et al.^18^ performed high-precision lung disease classification on chest X-ray maps using a custom MobileNetV2. The method first pre-processes the X-ray images in the dataset using CLAHE, and then feeds the processed images into several migration learning models such as InceptionV3^19^, AlexNet^20^, DenseNet^21^, VGG19^22^, and MobileNetV2^23^. On the pre-processed data, the fine-tuned MobileNetV2 achieved a classification accuracy of 96.97%. The analysis shows that applying transfer learning to convolutional filtering requires solving two problems: (1) The performance of these methods is comparable to wide/flat architectures e.g., VGG, but not to narrow/specific architectures e.g., GoogLeNet^24^, ResNet^6^. (2) Transfer learning is sometimes so powerful that it fails to guide the algorithms, making results unstable on certain datasets. Meanwhile, the above deep learning models require large number of parameters and more memory, which requires considerable inference time when classifying lung CT images in real time. Migration learning methods that introduce more information beyond a given medical dataset are based on non-medical images i.e., ImageNet datasets^25^, which may have performance limitations in the medical image domain^16^. Therefore, it is highly desirable to develop a deep network that can efficiently learn features from a limited set of lung CT image datasets without loss of performance.

In this work, most of the CNN models proposed for lung nodule detection and ImageNet pre-trained models (e.g. AlexNet, VGGNet, ResNet, etc.) use a single-branch architecture with layers stacked on top of each other. The convolutional layers in these networks are connected in a linear fashion and focus primarily on extracting features of the same proportion. However, these models suffer from significant deficiencies in the extraction of multiscale features, which have been shown to be very effective for complex image classification tasks, and therefore perform poorly in generalization in most cases. CT medical images do not show important features in some specific locations, but they can be found anywhere in the image. Therefore, when trained on such medical datasets, single-branch models are likely to miss features at finer levels of detail, thus affecting their classification performance. Several recent studies have demonstrated the benefits of using convolutional layers with multi-scale filters in CNN architectures^9,25^. Multi-scale filters help to extract features with different receptive field sizes, thus learning more detailed features and greatly improving the performance of the model. It is well known that CNN models with large number of parameters are widely used in computer vision tasks due to their excellent classification performance. However, they have a higher computational cost and a larger memory footprint compared to lightweight models, resulting in models that require longer inference and training times. When trained on small training datasets such as medical data, such models tend to suffer from overfitting problems. Moreover, such models are not suitable for real-time diagnosis of lung nodules. Therefore, we develop a lightweight deep integration network based on multi-scale interleaved fusion by incorporating multi-scale feature learning into the network model. It is able to extract useful features from a limited number of chest CT images while maintaining a small size and improving classification performance. The main contributions of this paper can be summarized as follows: (1) We propose a very lightweight deep network model (with a parameter of 0.7 M) combined with a multi-scale interleaved fusion learning module to classify the benign and malignant nature of lung nodules from chest CT scans, and call it MIFNet. (2) The proposed model consists of three multi-scale interleaved fusion learning (MIF) modules, where each module contains a mixture of convolutional layers (only 1 × 1 and 3 × 3 convolutional kernels) and shortcut links, which can be better exploited to efficiently integrate contextual information at adjacent resolutions using multilevel features, and to reduce the correlation between branch channels to improve the diversity of tandem integration components. (3) We have performed numerous experiments on the publicly available LUNA16 dataset and compared it with state-of-the-art methods to validate the superiority of MIFNet. In addition, we performed several ablation studies to find the most available set of hyperparameters that produce the best results.

## Material and method

### Datasets

We mainly used data from the Lung Image Database Consortium (LIDC-IDRI) image set^26^. As the LIDC dataset is very large (124 GB), we ended up using reformatted LUNA16^27^. This dataset excludes scans in the LIDC dataset with slice thicknesses greater than 2.5 mm and produces a total of 888 CT scans with images annotated with descriptive coordinates and ground truth labels. To be more suitable for clinical applications, a training database of images was created. The images were formatted as .mhd and .raw files. The header data is contained in the .mhd files and the .mhd files are read using the SimpleITK library, whereas the multidimensional image data is stored in the .raw files. There are approximately 200 images of size 512 × 512 × n in each CT scan, where n is the number of axial scans.

Statistically, there are a total of 551,065 annotations in this dataset, of which 1,351 are labelled as nodules and the rest are labelled as negative, so there is a large category imbalance in the data. To solve the problem of category imbalance, we under-sample most classes and increase some classes by rotating the image. In addition, training the deep network on the whole large image will cause problems such as increased computational cost and longer training time. Therefore, we crop the image by the coordinates provided in the annotations to produce a smaller image that is more suitable for evaluating the performance of the deep network. Since the annotations are represented by Cartesian coordinates, they need to be converted to voxel coordinates. At the same time, as the image intensities were defined in terms of the Hounsfield scale, the data had to be rescaled for effective image analysis. The adjusted data had 1 nodal map in every 6 images and was cropped to produce a 50 × 50 greyscale image. To further balance the number of training sample categories, we also increased the training set by rotating the images. The ultimate outcome consists of 6484 training samples and 1622 test samples.

### Network architecture and model development

**Fig. 1:**
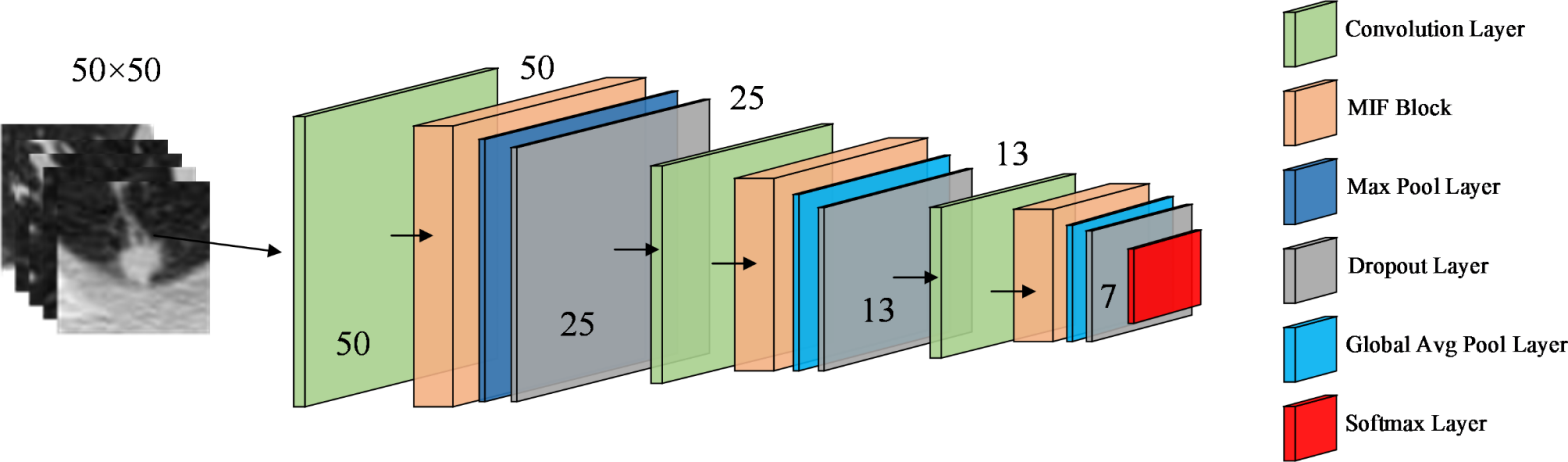
Illustration of the framework of the proposed multi-scale interleaved fusion integrated network classification model. It consists of three template modules. The first module follows a maximum pooling and dropout layer, while the remaining modules all follow a global average pooling and dropout layer. The output of the last module is fed into the classification layer to classify the benignity and malignancy of the lung nodules.

Figure 1 shows our proposed multi-scale interleaved fusion integrated network classification model for benign-malignant classification of lung nodules. The aim of this model is to design a lightweight CNN model with a multi-scale feature learning module that can learn essential features even from a limited number of chest CT images, while still maintaining a small size. The multi-scale interleaved fusion integrated network lung nodule classification model mainly consists of two parts: (1) A multi-scale interleaved fusion strategy, which uses three separate branches of different computational complexity for small and large patch markers, and interleaves and fuses these markers multiple times to complement each other, helps to extract features with different receptive field sizes and thus learn more detailed features. (2) Integrating the network structure, by constructing a template module using the network topology, and matching the number of feature maps in each grouped convolution to the number of feature maps in the convolutional layers of the template module, the deep network structure can be further simplified to improve computational efficiency. The implementation of the deep integrated network model consists of a stack of three template modules, each with the same number of network layers. The template modules aim to improve the receptive field while capturing multiscale features, as each point in the feature map space contains spatial information about its neighborhood and information about channel interaction. The multiscale interleaved fusion strategy can make better use of multilevel features to effectively integrate the contextual information of adjacent resolutions, reduce the correlation between branching channels to improve the diversity of tandem integration components, and greatly improve the performance of the model.

### Mini block

The MIF block consists of a stack of mini blocks, where the mini blocks also contain a sequence of three convolutional (Conv), batch normalization (BN) and ReLU layer. Consider a deep convolutional network with *l* layers, where the input image ${{\varvec{x}}_0}$ can be non-linearly transformed ${{\varvec{H}}_l}(\cdot )$ each time it passes through the convolutional layers and is defined as a composite function of three successive operations Conv, BN, ReLU. During the actual construction of the network, the convolutional layer uses 1 × 1 and 3 × 3 filters to capture finer detail features from the CT image. In this case, the padding values are kept ‘same’ in order to maintain a similar feature map size throughout the MIF block. The BN layer prevents model overfitting and facilitates the learning process, thus improving training convergence. Non-linearity can be introduced into the network learning process by adding a ReLU activation layer. In addition, we place the BN layer between the activation function and the convolution operation, so that the function of the forward convolution can be expressed mathematically as:

1 $$x_{{j^\prime }}^l = f\left( {{\text{BN}}\left( {\sum\limits_{j \in {M^l}} {x_j^{l - 1}} *f_{j{j^\prime }}^l} \right)} \right)$$

Where *l* is the number of layers in which this convolutional layer is located, ${\varvec{x}}_{{j^{\prime}}}^{l}$ denotes $j^{\prime}$ output feature map of the *l* layer, ${\varvec{f}}_{{jj^{\prime}}}^{l}$ is the convolutional kernel connecting *j* feature map of the $l - 1$ layer to $j^{\prime}$ feature map of the *l* layer, ${M^l}$ is the number of feature maps of the $l - 1$ layer, $f(\cdot )$ denotes the activation function ReLU, and * denotes the convolutional operation.

### MIF block

**Fig. 2:**
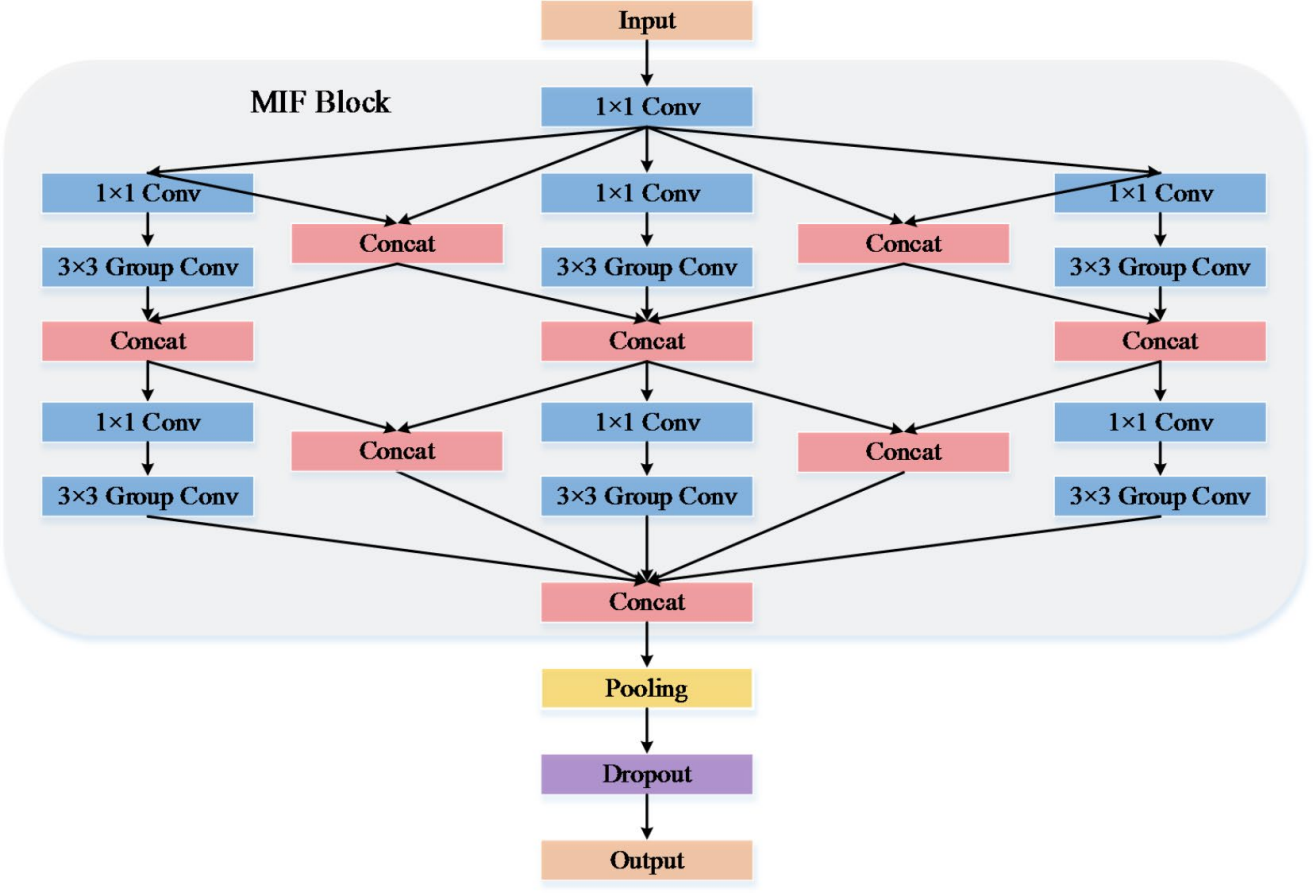
Schematic diagram of the proposed three-branch channel fusion template module.

The stacking of Mini block in the full MIF block is shown in Fig. 2. The MIF block is a multi-scale feature extraction module, which is an interleaving and fusion of three branch channels. When trained on the CT scan medical image dataset, the multi-branch structure and small convolutional kernel help to extract features with finer levels of detail, thus improving its classification performance. A deep network consisting of a stack of such modules can learn the features of different receptive fields by using a set of filters and improve its learning process, thus further improving its generalization performance on newly measured data. In order to learn multi-scale features, i.e., features with different receptive fields, filters of different sizes such as 3 × 3, 5 × 5, etc. are usually applied to the input data simultaneously. However, in contrast to small-sized filters, large-sized filters, while helpful in learning rough features such as the shape of the lung region, can also lead to large number of parameters being generated by the model. Specifically, a Conv layer with a filter size of 5 × 5 requires more than twice as many parameters as a Conv layer with a size of 3 × 3. Therefore, the construction of a lightweight network is particularly important for real-time diagnosis of the benign and malignant nature of lung nodules.

The mathematical expression for sensory field size calculation is described as follows, given that ${v_{in}}$ denotes the size of the input feature map, ${v_{out}}$ denotes the size of the output feature map, *k* denotes the size of the filter, *p* denotes the value of padding, and *s* denotes the step size. The output of the convolution operation can be expressed mathematically as:

2 $${v_{out}}=\frac{{{v_{in}}+2p - k}}{s}+1$$

Given *c* denotes a jump, the jump is an accumulation of step lengths, usually calculated by multiplying the step lengths of the previous layers. The jump in layer *l* can be expressed mathematically as:

3 $${c_l}={c_{l - 1}}*s$$

The formula for sensory field size is expressed as:

4 $${r_l}={r_{l - 1}}+(\eta - 1)*\prod\limits_{{\alpha =1}}^{{l - 1}} {{s_\alpha }}$$

where $\eta$ denotes the coefficient factor and ${r_l}$ denotes the size of the receptive field in layer *l*. From Eq. (4) it can be deduced that two conv slices with a filter size of 3 × 3 can be used to extract features with a receptive field size of 5 × 5.

The input feature maps in the MIF block are first passed through a scaling layer with a filter size of 1 × 1, and then a set of 3 × 3 filters and shortcuts are effectively combined to allow extraction of features with larger receptive fields while keeping the number of parameters small. The MIF block is a multi-scale feature extraction module that is repeatedly stacked three times throughout the proposed MIFNet structure. The design of this simple, highly modular network structure allows arbitrary scaling of transformations without specialized custom modules, while reducing the choice of hyperparameters and improving their learning process. To learn multi-scale features, the MIF block consists of a series of bottleneck modules, i.e., with the same topology, and follows the following rules: (1) When the feature map size output from a module is the same, this module shares the same hyper-parameters (e.g., width, convolutional kernel size, number of groupings, etc.). (2) When the feature map size is down-sampled, the width (i.e., the number of channels) of the module is doubled. Based on the above rules only one MIF block needs to be designed to identify all the modules, which results in a reduced model.

### Channel fusion for group convolutions

**Fig. 3:**
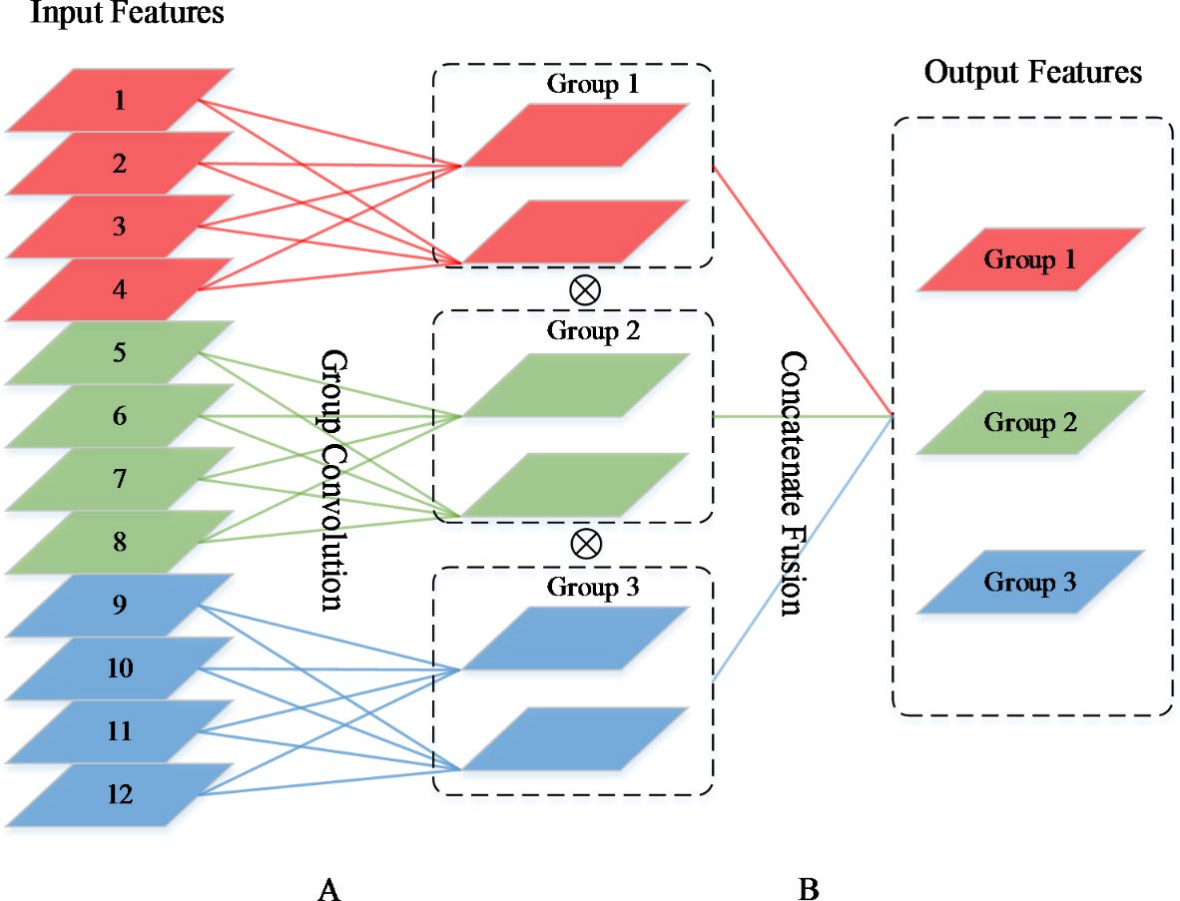
Channel fusion grouping schematic. **(A)** Group Convolution. **(B)** Group Concatenate Fusion. Sparse connectivity of channels is achieved by dividing the inputs into three non-intersecting groups, and the concatenate operation is used to fuse the different inter-channel information flow between different channels using the concatenate operation.

Most of the existing CNN models require large number of parameters and more memory space, and require considerable inference time when classifying CT images. Therefore, heavier models are not suitable for real-time diagnostics, especially when fast results are required. To address the imbalance between deep network expressiveness and computational efficiency, we introduce a packet fusion strategy in the Conv layer with a filter size of 3 × 3 to weaken the redundancy of the network. Figure 3(A) shows that group convolution reduces the computational cost by dividing the input features into several mutually exclusive groups and each group produces its own output. Clearly, the output of a particular group is only relevant to the inputs within that group. This impedes the flow of information between channels, which in turn weakens the expressive power of the network. Additionally, the greater the number of groups in a convolutional layer, the richer the information that can be encoded. However, as the number of input channels per group decreases and multiple group convolutions are stacked, a side effect occurs: the output of a given channel comes from only some of the input channels, which in turn weakens the expressiveness of the network to some extent. To solve this problem, we allow group convolution to obtain input data from different groups, as shown in Fig. 3(B). Specifically, we introduce group convolution only on 3 × 3 convolutional layers and perform channel fusion, as well as fusion of input information from different branch channels after each cascading group convolution, which then serves as input to the next layer, and so on. Note that multiple superimposed concatenation operations can significantly increase the feature map dimensions, which means that if the initial input feature dimensions are too large it will lead to an explosion in the number of channels. This would not be able to satisfy the complexity constraints and thus seriously affect the classification accuracy of the network. Therefore, during the actual construction of the MIFNet model, the dimension of the initial input image is generally chosen to be a smaller value.

### Overall MIFNet architecture

As shown in Fig. 1, the input image is first learned by down-sampling through a scaling layer, and this operation is repeated when the feature map size is halved. The model then sequentially contains three MIF blocks for efficient multi-scale feature extraction, each followed by a pooling layer to reduce the size of the feature map. Except for the first pooling layer, which uses the maximum pooling layer, all the others use average pooling with a dropout layer in series after each pooling layer. Finally, a classification layer with two neurons was introduced to classify CT images of lung nodules as benign or malignant using softmaxloss activation. In short, a highly lightweight deep integrated network model can be constructed by stacking MIF blocks with only 0.7 M parameters and multi-scale feature learning architecture, which performs well in distinguishing between positive and negative lung nodule samples. Table 1 shows the network configuration of the proposed MIFNet.

**Table 1:** MIFNet architecture configuration.

Layers	Output Size	Description
Scaling Layer	50 × 50	1 × 1 conv, 16, stride 1
MIF Block	50 × 50	$$\left[ \begin{gathered} 1 \times 1,4 \\ 3 \times 3,16 \\ \end{gathered} \right] \times 3$$ / $$\left[ \begin{gathered} 1 \times 1,4 \\ 3 \times 3,16 \\ \end{gathered} \right] \times 3$$ / $$\left[ \begin{gathered} 1 \times 1,4 \\ 3 \times 3,16 \\ \end{gathered} \right] \times 3$$
Pooling Layer	25 × 25	2 × 2 max pool, stride 2, dropout 0.1
Scaling Layer	25 × 25	1 × 1, 32, stride 1
MIF Block	25 × 25	$$\left[ \begin{gathered} 1 \times 1,8 \\ 3 \times 3,32 \\ \end{gathered} \right] \times 3$$ / $$\left[ \begin{gathered} 1 \times 1,8 \\ 3 \times 3,32 \\ \end{gathered} \right] \times 3$$ / $$\left[ \begin{gathered} 1 \times 1,8 \\ 3 \times 3,32 \\ \end{gathered} \right] \times 3$$
Pooling Layer	13 × 13	2 × 2 average pool, stride 2, dropout 0.2
Scaling Layer	13 × 13	1 × 1, 64, stride 1
MIF Block	13 × 13	$$\left[ \begin{gathered} 1 \times 1,16 \\ 3 \times 3,64 \\ \end{gathered} \right] \times 3$$ / $$\left[ \begin{gathered} 1 \times 1,16 \\ 3 \times 3,64 \\ \end{gathered} \right] \times 3$$ / $$\left[ \begin{gathered} 1 \times 1,16 \\ 3 \times 3,64 \\ \end{gathered} \right] \times 3$$
Pooling Layer	7 × 7	2 × 2 average pool, stride 2, dropout 0.3, FC, softmaxloss

### Algorithm steps

In summary, the main steps of the proposed MIFNet algorithm can be summarized in Algorithm 1.

**Table:** 

Algorithm 1: Proposed MIFNet	
**Inputs**: input image ${{\varvec{x}}_0}$, the control parameters *l* and ${M^l}$. 1. The LUNA16 dataset is preprocessed to obtain the input images ${{\varvec{x}}_0}$, with each image being with size 50 × 50. 2. The feature mapping matrix is obtained ${\varvec{x}}_{{j^{\prime}}}^{l}$ through Eq. (1). 3. The input image ${{\varvec{x}}_0}$ passes through the convolutional layer both can achieve a nonlinear transformation ${{\varvec{H}}_l}(\cdot )$. 4. The MIFNet architecture was constructed according to Table 1. 5. Set the maximum number of iterations to 50 epochs. 6. Batch size set to 32. 7. Initialize learning rate set to 0.001. 8. The ADAM optimization algorithm was used to train MIFNet. **Output**: The predicted class labels for testing samples.	

## Results

### Experiments setup

We used an available reformatted version of LUNA16 and created a database of images for training. The implemented software environment is Windows 10, the hardware environment is Intel Core i7 and a Nvidia GeForce GTX 1660 GPU, the programming development environment is CUDA 8.0, and the deep learning framework is MATLAB. To further evaluate the effectiveness of the proposed model, we compared the results with pre-trained ImageNet deep models and state-of-the-art methods. In addition, we carried out various ablation experiments to help understand how different factors affect the MIFNet model. Factors analyzed included the number of volume integration groups, the number of branch channels and the choice of optimizer. To evaluate the proposed MIFNet as well as other existing models, we used different evaluation metrics such as accuracy (Acc), F1 score (F1), precision (Prec), sensitivity (Sens), specificity (Spec). In order to better train the network, Xavier weight initialization was used in this study, and other hyperparameters were set as follows: 50 training epochs, batch size of 32, and an initialized learning rate of 0.001.

### Analysis of group numbers

**Fig. 4:**
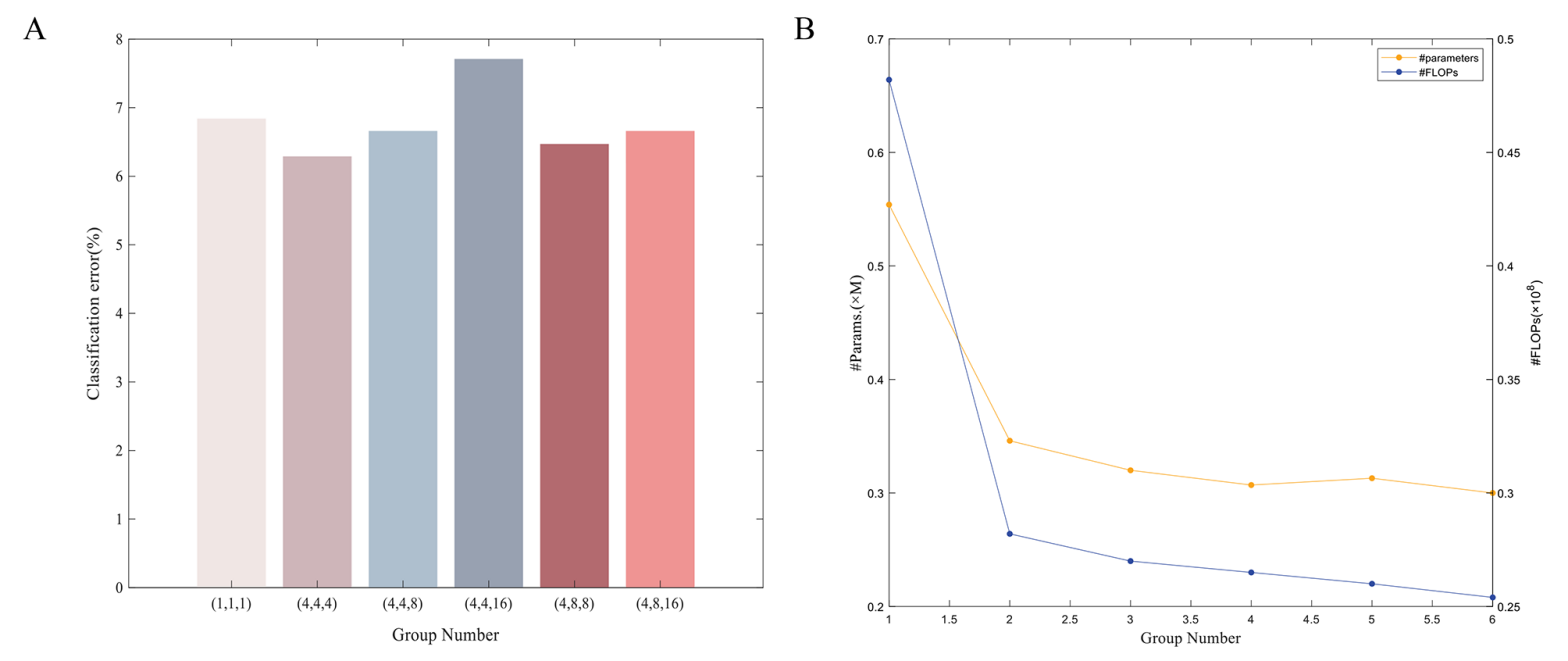
Performance comparison of MIFNet with different groups number on LUNA16. **(A)** Performance histogram of error rate and number of groups for MIFNet. **(B)** Comparison of the number of subgroups as a function of parameters and flops learned by MIFNet.

To assess the impact of various grouping numbers on enhancing the computational efficiency of lung nodule detection using MIFNet, we chose different grouping configurations and conducted experiments on the reconstructed LUNA16 dataset. First, the pooling layer is used as the transition layer of the network architecture and the template modules between adjacent transition layers have the same number of groups. Then the MIFNet at layer 22 is used as the baseline and the number of packets is set to vary between 1 and 16. This means that the computational complexity of the model varies with the number of groups. Figure 4 shows the performance comparison of the proposed MIFNet model on the LUNA16 dataset with respect to the number of subgroups, where the classification error rate is shown on the left and the number of parameters and FLOPs are shown on the right. Through experiments, we find that the performance of lung nodule classification can be effectively improved by introducing a grouped convolutional channel fusion strategy in deep integration networks. As the number of packets increases, both the number of network parameters and FLOPs show a decreasing trend and are compressed by almost 50%. It is worth noting that, as shown in Fig. 4(B), when the number of subgroups is 4, the number of network parameters and FLOPs are already at a relatively low position, and as the number of subgroups further increases, it is not obvious to reduce the number of parameters and improve the computational efficiency, but rather to waken the expressiveness of the network. In particular, the lowest classification error rate was obtained when the number of groupings of the three template modules of the network was (4,4,4). Through further analysis, we found that the three template modules with the pooling layer as the transition layer have the same FLOPs. Therefore, the following experimental analyses will use the grouping number of (4,4,4) without compromising performance.

### Optimizer options

**Fig. 5:**
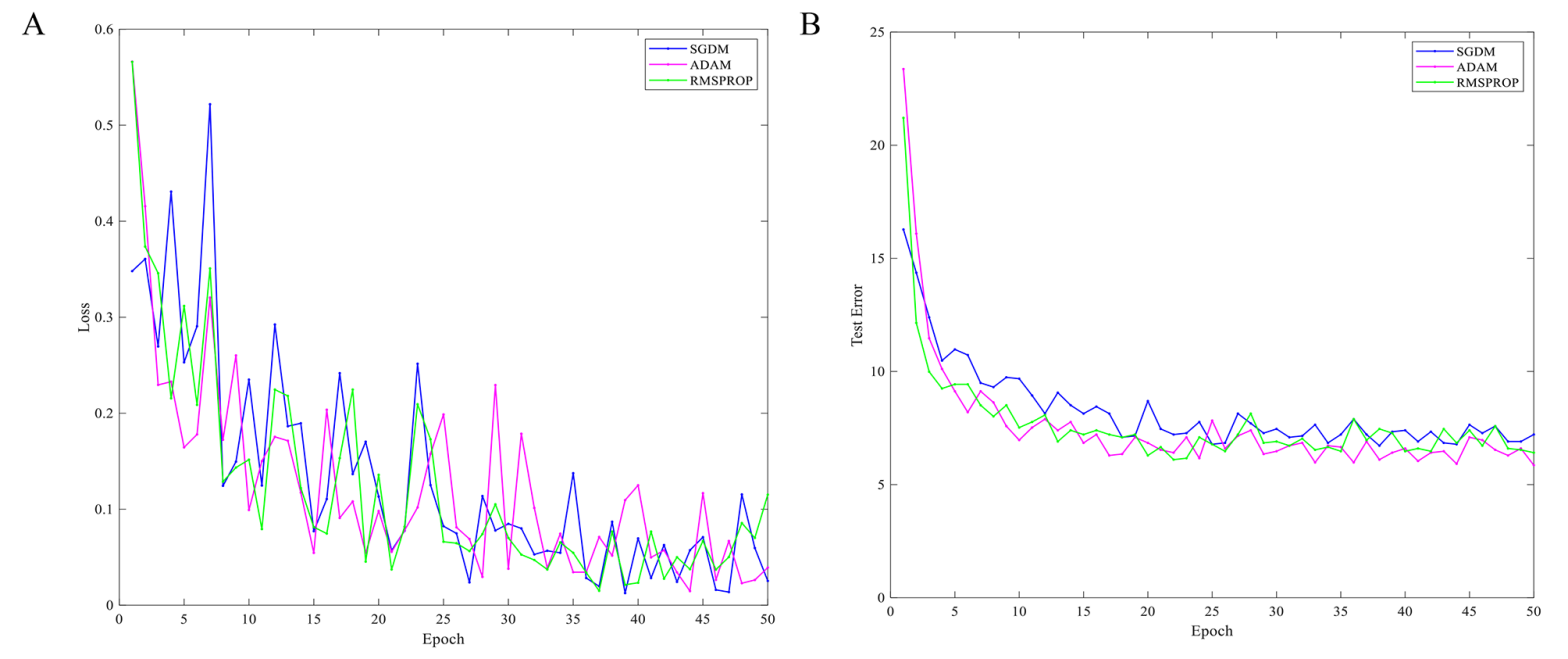
Optimization algorithm loss function and classification error rate curves. **(A)** Loss function curves. **(B)** Classification error rate curves.

The gradient descent algorithm is renowned for being the most widely utilized optimization method among various machine learning algorithms. However, it is frequently employed as a black-box optimizer, which has its limitations. In practice, therefore, the choice of algorithm needs to be tailored to specific requirements, as different approaches may be more suitable depending on the circumstances. In this section, we validate the convergence and classification error rate of the SGDM, ADAM and RMSPROP algorithms using a 22-layer MIFNet as a benchmark over 50 training sessions. The loss function used in the experiments is the softmax cross-entropy loss, and to compare the performance more accurately, the loss function and classification error rate curves of the three algorithms are plotted in Fig. 5. Throughout the training process, it is evident that the values of the loss functions and the classification error rates for the three optimization algorithms exhibit a consistent trend of improvement, indicating a decrease. However, in the initial phase of training, ADAM and RMSPROP have higher loss function values compared to SGDM, and the corresponding classification error rates are in line with them. As the number of iterations increases, the convergence curve of the ADAM algorithm shows large oscillations compared to SGDM and RMSPROP, especially between 25 and 45 epochs. In the final stage of training, a lower classification error rate is obtained because ADAM has a lower loss function value. The main reason for this is that the second-order momentum of ADAM changes with the number of iterations, which makes the learning rate of the algorithm not monotonically decreasing and generates oscillations in the later stages of training, which in turn leads to subsequent oscillations in convergence. Therefore, the ADAM optimization algorithm is used to train subsequent experiments.

### Number of fusion channels

We investigate the impact of varying the number of fusion channels on both the performance and computational efficiency of the proposed network. To empirically assess this, we evaluate the network’s performance using different numbers of fusion channels, with MIFNet configured with a grouping number of (4,4,4) serving as the experimental baseline network. As shown in Table 2, the relationship between the computational complexity of the network and the classification accuracy for different numbers of fusion channels as the network layers deepen, where “-” indicates that the corresponding computation was not performed due to exceeding the computer memory. It can be observed that when MIFNet maintains the same depth, its computational complexity exhibits a notable exponential increase with the rising number of fused channels, while the classification accuracy also improves accordingly. Specifically, with a fusion of 2 channels, MIFNet attains the highest classification accuracy of 94.82%. This indicates that simply increasing the number of fusion channels does not improve the representational capacity of the proposed network, but rather leads to a significant consumption of computational resources. Therefore, to optimally balance the trade-off between network expressiveness and computational efficiency, MIFNet with a fusion channel number of 2 (C = 2) is adopted as the foundational network for subsequent experimental analyses.

**Table 2:** Comparison of MIFNet performance with different number of fusion channels.

Depth	FLOPs (×10^8^)				Acc (%)			
	MIFNet (C = 2)	MIFNet (C = 3)	MIFNet (C = 4)	MIFNet (C = 5)	MIFNet (C = 2)	MIFNet (C = 3)	MIFNet (C = 4)	MIFNet (C = 5)
10	0.052	0.095	0.131	0.167	93.59	93.53	93.83	93.40
22	0.282	0.974	1.161	1.614	94.64	94.45	94.2	93.71
28	0.559	2.222	3.698	5.434	94.51	94.27	93.96	94.27
34	1.099	5.773	-	-	94.51	93.77	-	-
40	2.169	-	-	-	**94.82**	-	-	-
46	4.369	-	-	-	94.08	-	-	-

### Deeper and wider networks

**Fig. 6:**
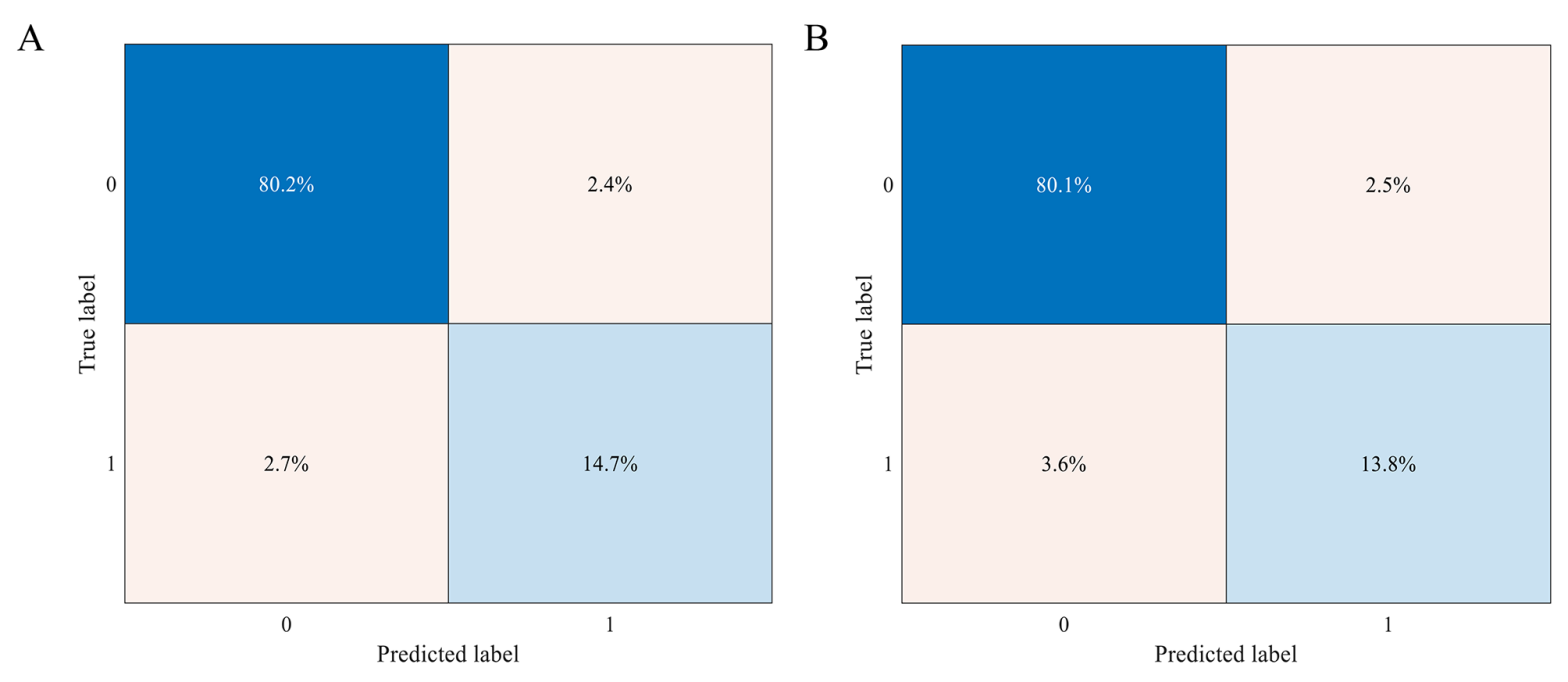
Confusion matrix obtained by MIFNet on the LUNA16 dataset. **(A)** MIFNet-40(4,8,16). **(B)** MIFNet-46(4,8,16).

Using MIFNet with a configuration of 4 groups per layer (4,4,4) and 2 fusion channels as our experimental baseline network, we investigated how the depth and width of the network affects its performance and computational efficiency. Table 3 shows that MIFNet’s performance varies as the depth increases, given the feature map dimensions of (4,8,16) for the three template modules in the network. The network depth shows a direct correlation with the number of parameters (space usage), model size (memory usage), and computational complexity (execution speed). Conversely, it exhibits an inverse relationship with classification accuracy. As the number of layers deepens, the classification accuracy gradually increases and starts to decrease after reaching the extreme value. When the feature map dimensions of the three template modules are extended to (8,16,32), Table 4 shows the performance comparison of MIFNet with increasing network depth. It is evident that the classification accuracy of the model can be significantly enhanced by increasing the network width, which essentially means expanding the network size. However, as the size of the structure increases, the network also generates large number of parameters, which in turn increases the redundancy of the network. As shown in Table 4, for network depths of 22 and 28 layers, the network classification error rate also increases by about 6% as the number of parameters increases. Figure 6 shows the confusion matrices of our proposed MIFNet-40(4,8,16) and MIFNet-46(4,8,16) on the test set. Both confusion matrices show that the percentages of false positives and false negatives are low. However, MIFNet-40(4,8,16) exhibits a lower combined percentage of false positives and false negatives compared to MIFNet-46(4,8,16), indicating higher and more balanced prediction accuracy for the MIFNet-40(4,8,16) model. The experimental analyses above highlight that enhancing either the depth or the width of the network leads to improved classification accuracy for the model. Specifically, by adjusting these parameters, we observe a positive impact on the model’s performance, demonstrating the significance of network depth and width in achieving better classification results. However, a relatively small and deep network for the proposed network is better than a large and wide network, which is consistent with the lightweight network model we designed.

**Table 3:** Comparison of MIFNet performance with different number of depths.

Depth	Params. (M)	Size (MB)	FLOPs (×10^8^)	Acc (%)
10	0.017	0.064	0.052	93.59
22	0.091	0.346	0.282	94.64
28	0.18	0.687	0.559	94.51
34	0.356	1.356	1.099	94.51
40	0.70	2.681	2.169	**94.82**
46	1.394	5.319	4.369	94.08

**Table 4:** Comparison of MIFNet performance with different number of widths.

Depth	Params. (M)	Size (MB)	FLOPs (×10^8^)	Acc (%)
10	0.043	0.164	0.142	93.59
22	0.218	0.831	0.722	94.33
28	0.425	1.62	1.405	94.08
34	0.825	3.148	2.721	94.64
40	1.613	6.152	5.303	94.08
46	3.15	12.016	10.418	**94.94**

### Comparison with ImageNet pretrained models

Deep transfer learning has been shown to be an effective learning strategy that can extract representative imaging biomarkers from lung CT images for lung nodule classification and reduce training time. To verify the advantages of the proposed MIFNet in lung nodule classification, we fine-tuned the pre-trained ImageNet CNN architecture under the same experimental dataset distribution and experimental environment, and further compared the model performance. To highlight the overall trend, the classification results of the proposed method are shown in bold. The specific experimental results are shown in Table 5, where ‘-’ indicates that there is no relevant description in the literature. It can be observed that most ImageNet pretrained models are very large, with network parameters in millions and memory usage in MB, such as VGG-19 require 143.6 M parameters, ResNet-101 requires 44.6 M parameters, and Inception V4 requires 55.8 M parameters. For small networks, ShuffleNet requires 1.4 M, EfficientNet B0 requires 5.3 M, and SqueezeNet requires 1.2 M. Compared to other methods, our proposed 40-layer MIFNet (4,8,16) model requires only 0.7 M parameters (approximately 0.49% of VGG-19, 1.58% of ResNet-101, 1.26% of Inception V4, and 50.21% of ShuffleNet) and achieves a classification accuracy of 94.82%. It can be observed that although our model has the lightest architecture of all techniques, it outperforms all ImageNet models on the LUNA16 dataset, which is consistent with the performance of other ImageNet pre-trained models and our proposed method. It is worth noting that the 46-layer MIFNet(8,16,32) by extending the network depth and width did not achieve the best performance in other evaluation metrics although it obtained the highest classification accuracy. Therefore, the 40-layer MIFNet(4,8,16) is more suitable for real-time classification using CT images.

**Table 5:** Comparison of MIFNet performance with ImageNet pre-trained CNN models.

Method	Params. (M)	Size (MB)	Depth	Acc (%)	F1 (%)	Prec (%)	Sens (%)	Spec (%)
VGG-16^22^	138.3	515	16	93.29	95.45	91.53	**99.72**	53.27
VGG-19^22^	143.6	535	19	93.99	95.73	92.29	99.45	57.94
ResNet-18^6^	11.6	44	18	91.44	96.15	93.24	99.26	63.55
ResNet-50^6^	25.5	96	50	93.14	96.56	94.68	98.52	71.96
ResNet-101^6^	44.6	167	101	94.37	96.66	94.45	98.98	70.56
GoogleNet^24^	6.9	27	22	94.29	96.55	94.84	98.34	72.90
Inception V3^19^	23.8	89	48	90.13	96.54	93.97	99.26	67.76
Inception V4^28^	55.8	209	164	94.22	96.47	95.61	98.52	77.10
DenseNet-201^21^	20.0	77	201	91.29	97.28	95.96	98.71	78.97
MobileNet V2^23^	3.5	13	53	91.06	96.23	93.55	99.08	65.42
ShuffleNet^29^	1.4	5.2	50	91.13	96.75	94.77	98.80	72.43
Xception^30^	22.9	85	71	88.90	96.12	93.01	99.45	62.15
EfficientNet B0^31^	5.3	19.9	82	87.66	96.42	93.65	99.35	65.89
DarkNet-19^32^	20.8	78	19	91.52	96.69	94.93	98.52	73.36
DarkNet-53^33^	41.6	155	53	91.67	96.45	93.96	99.08	67.76
SqueezeNet^5^	1.2	5.2	18	91.44	95.28	91.93	98.89	56.07
NasNet-Mobile^34^	5.3	20	-	88.28	96.27	93.71	98.98	66.36
MIFNet(4,8,16)	**0.7**	**2.681**	40	94.82	**97.34**	**96.74**	97.10	**84.75**
MIFNet(8,16,32)	3.15	12.016	46	**94.94**	96.42	95.73	97.01	79.43

### Comparison with existing methods

To highlight the superior performance of our proposed method MIFNet, we conducted a comprehensive analysis comparing it with other leading state-of-the-art methods^35–41^. The results of this comparison are presented in Table 6, showcasing the distinctive advantages and innovative capabilities of MIFNet in terms of performance. The experimental results show that compared with other methods, our proposed method achieves the highest F1 value and accuracy index, which are 97.34% and 96.74%, respectively. In addition, MIFNet achieved the second highest accuracy and sensitivity. In computer-aided diagnosis, sensitivity, which refers to the true positive rate, plays a crucial role in accurately identifying patients with malignant lung nodules. Meanwhile, our proposed MIFNet model boasts the highest F1 value, indicating that it has achieved an optimal balance between sensitivity and specificity. In other words, MIFNet possesses the ability to accurately classify the majority of lung nodules, thereby significantly alleviating the workload of physicians. Overall, with the exception of^35^ and ^37^, the methodology proposed in this paper demonstrates significant advantages and outperforms most studies across all evaluation metrics. Notably, MIFNet boasts an accuracy nearly 0.9% higher than^37^ and a sensitivity 5.0% higher than^35^, underscoring its broad applicability and expertise in advanced lung nodule classification. This outstanding performance is mainly due to its multi-scale feature learning capability and a very lightweight network design strategy that exploits multilevel features to efficiently integrate contextual information at adjacent resolutions, and reduces the correlation between branch channels to improve the diversity of tandem integration components. This not only allows more detailed features to be learned to improve the accuracy of the model, but can also be applied to real-time clinical diagnostics, demonstrating the robustness and effectiveness of the model.

**Table 6:** Performance comparison with existing works.

Method	Acc (%)	F1 (%)	Prec (%)	Sens (%)	Spec (%)
VGG + ResCon(3D) & Focal loss & Fine-tune^35^	**95.37**	93.04	93.62	92.48	**96.83**
Ensemble of 3D Dual Path Networks^36^	90.24	90.45	88.85	92.04	88.94
FractalNet + CNN^37^	94.06	88.96	81.78	**97.52**	86.76
Deep3DSCan^38^	88.33	88.52	90.00	87.10	89.66
NASLung^39^	90.77	89.29	93.58	85.37	95.04
LSTM + VGG16^40^	90.0	94.0	97.01	91.17	73.17
sGBN^41^	90.85	88.89	92.01	86.04	93.38
MIFNet(4,8,16)-40	94.82	**97.34**	**96.74**	97.10	84.75

### Visualization of MIFNet model predictions

**Fig. 7:**
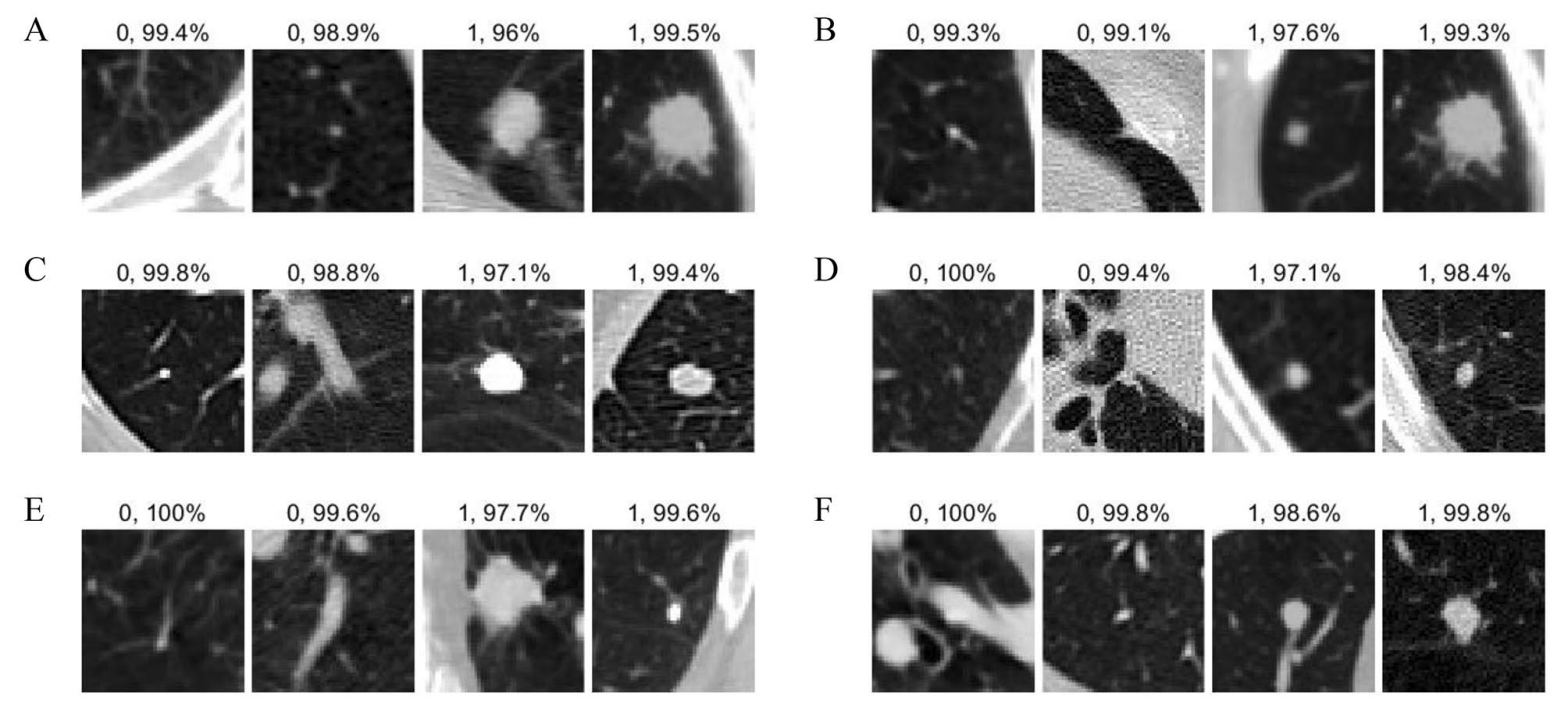
The visualization results of predicting the benign and malignant nature of lung nodules, with numbers representing the category labels and the predicted confidence. **(A)** EfficientNet B0. **(B)** NasNet-Mobile. **(C)** MobileNet V2. **(D)** ShuffleNet, **(E)** SqueezeNet. **(F)** MIFNet-40.

To provide a more intuitive understanding of the performance of the proposed network in classifying lung nodules, we also provide examples of visual explanations of the model’s predictions. As shown in Fig. 7, four example validation images and predicted labels are displayed, along with the predicted probabilities of these images. From top to bottom, the rows in Fig. 7 show the prediction results of a representative selection of EfficientNet B0, NasNet-Mobile, MobileNet V2, ShuffleNet, SqueezeNet and MIFNet-40 models. From left to right, the image columns are two columns of benign and malignant lung nodules, respectively. As can be seen in the figure, benign and malignant lung nodules have similar prediction probabilities for all models, indicating that our recreated LUNA16 dataset effectively solves the category imbalance problem. In addition, the prediction probabilities of the MIFNet-40 model in these example images are significantly higher than those of the other algorithms, suggesting that MIFNet can further improve the learning performance of the deep network while scaling down the model.

## Discussion

In this study, we propose a lightweight interleaved fusion integrated network with multiscale feature learning, which aims to extract multiscale features from a limited number of CT images using a small network for accurate and timely benign and malignant classification of lung nodules. Using three separate branches of different computational complexity for small and large patch markers, and interleaving and fusing these markers multiple times to complement each other, helps to extract features with differently sized receptive fields and thus learn more detailed features. The deep network structure can be further simplified to improve computational efficiency by constructing a template module using the network topology and matching the number of feature maps in each grouping to the number of feature maps in the convolutional layers of the template module. We demonstrate that no significant degradation in classification performance is observed when the proposed model does not require large number of parameters and carefully annotated data, which will reduce the computational cost and memory requirements for models in this research area.

Deep learning algorithms have been highly successful in lung CT image recognition tasks^3,4,8,9,17,18^. However, while previous studies have successfully reduced the complexity of the network, they have overlooked certain spatial information, which is crucial for accurate lung nodule classification and thus poses a disadvantage. Furthermore, the use of data augmentation techniques only focuses on the target task on a given dataset, as the dataset of pathologically confirmed lung nodules is largely missing and highly unbalanced in terms of benign and malignant distributions, and there is a limit to improving model performance by only fine-tuning the model itself or augmenting datasets with unbalanced distributions. While transfer learning strategies suggest that features learned to solve a particular task are also useful for tasks in other domains, transfer learning methods that introduce more information beyond a given medical dataset are based on non-medical images, which may have limitations for performance in the medical image domain. This study demonstrates that the proposed multi-scale interleaved fusion integration network excels at effectively integrating contextual information across adjacent resolutions by leveraging multilevel features. Additionally, it minimizes the correlation between branch channels and enhances the diversity of tandem integration components. Table 4 shows that MIFNet achieves a classification accuracy of 94.82% with just 0.7 million parameters, highlighting the superiority of our proposed method compared to traditional approaches. Furthermore, as evident from the confusion matrix and the visualized prediction results, our method demonstrates superior and balanced performance across all categories. Our method can be applied not only to classifying lung nodules as benign or malignant, but also to various other computer-aided medical image analysis tasks, thereby offering significant clinical applications and demonstrating its valuable utility in the field of medical image analysis.

## Conclusion

This paper proposes a lightweight integrated network with multi-scale learning modules (MIFNet) to solve the challenging problem of diagnosing benign and malignant lung nodules in chest CT images. Specifically, we develop a novel multi-scale staggered fusion strategy that can better exploit multi-level features to effectively integrate contextual information from adjacent resolutions, reduce the correlation between branch channels, and improve the diversity of tandem integration components. In addition, the model size can be effectively reduced by repeatedly stacking the same topology and keeping the number of feature maps in each MIF block convolutional layer consistent. The proposed MIFNet is validated and evaluated on the reconstructed LUNA16 dataset, and ablation experiments are performed. The experimental results show that MIFNet achieves good classification performance with a small number of model parameters and memory footprint, making it suitable for real-time diagnosis of benign and malignant lung nodules.

### Limitations of the study

There are several limitations to this study. First, although our method performs better with limited data, only rotated images were used to augment the dataset when balancing the number of training sample categories. Therefore, different data augmentation strategies should be further selected to improve the classification results. As our dataset was reconstructed based on public data, there may be some poor-quality lung CT images in large-scale real-world clinical classification scenarios, which may affect the performance of the model. In addition, the model does not fully use the valuable attribute prior knowledge of lung CT images for semantic reasoning, which makes it difficult to be understood and applied by clinical radiologists, and future research should focus on developing models with interpretability.

## Data Availability

The original image files are not publicly available due to their containing information that could compromise the privacy of research participants. The datasets generated and analyzed for this study are available from the corresponding author (JQT), upon reasonable request.
